# 

**Published:** 1850-06

**Authors:** 


### WESTERN JOURNAL

OF

### MEDICINE AND SURGERY.

EDITORS.

---

LUNSFORD P. YANDELL, M. D.
PROFESSOR OF PHYSIOLOGY AND PATHOLOGICAL ANATOMY IN THE UNIVERSITY OF LOUISVILLE.

AND

THEODORE S. BELL, M.D.

### INDEX TO VOL. V.

### [THIRD SERIES.]

A

Abscesses in the brain............245
Abscesses, fistulous..............497
A case of chronic pneumonia, by Dr. D. W. Yandell............312
Alabama, typhoid fever in........182
Albuminaria.....................192
Alcoholic extracts...............275
American medical association....273
American surgery...............179
Ancient medical literature........393
Anderson's case of purpura......470
Anemic and other affections, employment of manganese in.....259
Annual of scientific discovery,....433
Anthrax, case of................423
Anus, treatment of fissures in....264
A practical treatise on diseases of children, by D. Francis Condie, M. D. ....................... 92
A professorial Thersites...........453
Arsenical poisoning, Siliman's case of....................... 49

B

Bandage, the in the treatment of fractures, ulcers, gunshot wounds hemorrhage, &c, &c., by T. S Bell, M D.................. 93
Bandage, the in the treatment of fractures, &c..................390
Baron Larrey's method of treating ulcers caused by congelation... 97
Bartlett Prof. E on the life, &c., of Wm. Charles Wells, M. D... 22
Bartlett's Prof. address........... 91
Bartlett Professor on edematous laryngitis......................209
Baynton's method of treating ulcers...........................409
Beck's historical sketches of the state of medicine in the American colonies, &c...............542
Beds for the sick................501
Bell on the bandage.............. 93
Bell's T. S. remarks on the reports in the London Lancet on fractures, hemorrhages, &c.....477
Bell's miscellaneous matters of practical medicine...............500
Bell John on the bandage....... 99
Bell's John description of the mode of applying the bandage..139
Bigelow's introductory lecture....455
Brain, abscesses in..............245
Brodie on dressing ulcers........422

C

Carbuncle, treatment of..........424
Cases of Purpura............... 13
Cases at the Louisville hospital, by Dr. D. W. Yandell..........241
Case of malignant tumor........448
Cholera, cause of...............275
Clinical lectures.................455
Coagula, fibrinous of the heart, Yandell Dr. D W. on..........369
Colleges in the U. S.............270
Constitutional treatment of ulcers.421
Cornea, opacities of.............443
Critchett on ulcers..............399
Critchett on water dressings......417
Critchett on method of strapping ulcers.........................419
Cui Bono?...................... 90
Cutler on the bandage........... 96
Cystic Oxide....................196

D

Death, Dr. Dowler on............545
Demonstrative midwifery........459
Demonstrative midwifery........539
Descendants of Balaam's beast....397
Diabetes.......................189
Diet, in the cure of malignant tumor.448
Division of ulcers...............402
Dowler on death................545
Drake's treatise.................543
Dysentery, Sneed on............ 1
Dysentery and diarrhea of fever, treatment of....................258

E

Edematous laryngitis, Professor Bartlett on....................209
lesions.............210
symptoms...........213
causes.............218
varieties & forms. of................221
march, duration, and terminations.....222
mortality and prognosis.............223
diagnosis...........223
theory.............225
treatment..........226
definition..........237

Bartlett on history and bibliography.......................238
Emison's case of traumatic tetanus—recovery..................317
"Enquirer" on the bandage......408
Epidemic dysentery, by Dr. Wm. C. Sneed.................... 1
Epidemic puerperal peritonitis....461
Esophagus, extraction of foreign bodies from..................262
Experiments on the use of salt....532
External hemorrhoids, treated by potassa fusa..................268
Extracts, alcoholic...............275
Eve's Prof. introductory lecture, notice of..................366

F

Fever, typhoid in Alabama........182
Fever, typhus ....................258
Fibrinous coagula of the heart, Dr. D. W. Yandell on.........369
Fissures of the anus, treatment of.264
Fistulous abscesses...............497
Fracture of the skull, Swain's case of 473
Fractures, the London Lancet on.477

G

Gangrenous ulcer, remarks on by T. S. Bell.....................485
Gastritis, case of reported by Dr. D. W. Yandell.................241
Gordon's case of rupture of the ilium and omentum.............142
Gordon's lectures on water, with a number of cases...............277

H

Haskins on the diagnostic value of the urine...................185
Heart, coagula of Yandell on.....369
Heberden on the bandage.........407
Heister on the bandage...........395
Heister on the bandage in fistulous cases....................405
Heister on the bandage in fractures 406
Hemorrhage post-partum, fatal....300
Hemorrhoids, externally treated by potassa fusa...............268
Hemorrhoids, new instrument for operating on..................441
Hewett's case of malformation of the uterus, with fatal post-partum hemorrhage ..................300
Historical sketch of the state of medicine in the American colonies, &c.........................542

I

Impropriety of frequent operative interference in midwifery......441
Inflammation of the urinary passages 200
Instruction, summer..............548
Intestinal obstruction in the colon, operation for.................505
Iodine, solution of in the treatment of nevus.......................262

J

Jenner on typhoid, typhus, and relapsing fever..................535
Journal, St. Louis Medical.......548

K

Kenney on epidemic puerperal peritonitis.....................461
Kentucky surgery................178
Laryngitis edematous, Prof. Bartlett on.......................209
Leucorrhea, new remedy for......442
Louisville marine hospital, cases at, reported by Dr. D. W. Yandell........................241

M

Macartney on water dressings for ulcers ........................417
Maclise on the anatomical phenomena of fractures............133
Maclise on splints................134
Malignant tumor.................448
Manganese in anemic and other affections ....................259
Medical men's per centage on prescriptions ....................181
Medical society of Montgomery county Tenn.................182
Medicine veterinary..............458
Medical Journal, St. Louis........548
Meeting of the American medical association, at Cincinnati......544
Midwifery impropriety of frequent operative interference in.......441
Midwifery, demonstrative........459
Morbus brightii ..................197
Mortality from injuries......102, 103

N

Nevus treated by solution of iodine .........................262
New instrument for operating in hemorhoids ...................441
New remedy for leucorrhea......442
Nitrate of silver in salivation......266
Nitrate of silver in lacerated wounds of the face, &c.........500

O

Obituary ........................276
O'Halleran on adhesive inflammation ..........................106
On beds for the sick.............501
On the identity or non-identity of typhoid fever, typhus fever, and relapsing fever................535
Opacities of the cornea...........443
Opening made in the colon......505
Operative interference in midwifery..........................441
Oxalate of lime..................195

P

Paré's ointment for gun-shot wounds....107
Pathological changes that follow section of the sciatic nerve....531
Per-centage on prescriptions....181
Pharmacy's society of premium....248
Philosophy of the bandage....124
Phosphatic deposits....194
Pirtle's case of complicated disease....320
Pneumonia, chronic — Yandell's case of....312
Poisoning by arsenic, a case reported by Prof. Silliman.... 49
Postage on scientific journals....183
Postponed items....366
Potassa fusa in the treatment of external hemorrhoids....268
Premium offered by the society of pharmacy of Paris....248
Purpura, case of by Dr. B. F. Wendell.... 13
Purpura, case of by Dr. N. B. Anderson....470
Purulent secretions, effects on the constitution....493
Remarkable complication of diseases, by Dr. C. Pirtle....320
Reviews—
A treatise on the diseases of the bones. By Edward Stanley, F. R. C. S., President of the royal college of surgeons of England, and surgeon of St. Bartholomew's hospital.... 58
The three kinds of cod liver oil. By L. J. de Jongh, M. D., of the Hague.... 86
A critical analysis of the review of Prof. B. W. Dudley's article on aneurism....144
Lectures on the diseases of infancy and childhood. By C. West, M. D., physician to the royal infirmary for children, &c....163
On the diseases of children. By Fleetwood Churchill, M. D., M. R. I. A., hon. fellow of the college of physicians, Ireland, &c....163
A practical treatise on the diseases of children. By D. Francis Condie, M D., secretary of the college of physicians, &c....163
Principles of human physiology; with their chief applications to pathology, hygiene, and forensic medicine. By Wm. Carpenter, M. D., F. R. S. F. G. S., examiner in physiology in the university of London....168
The transactions of the American medical association, vol. 2..174
The American Journal of Science and Arts. By Prof. B. Silliman, LL. D., M. D., &c., Prof. B. Silliman, Jr., A. M., M. D., &c., and James D. Dana, A. M....252
Braithwaite's retrospect of practical medicine, and Rankin's half yearly abstract of the medical sciences....257
On the treatment of gun-shot wounds. By B. W. Dudley, M. D., professor of surgery in Transylvania university.
On the use of the bandage in gun-shot wounds, etc. By B. W. Dudley, M. D., professor of anatomy and surgery in Transylvania university....324
A theoretical and practical treatise on midwifery, including the diseases of pregnancy and parturition. By P. Cazeaux, adjunct professor in the faculty of medicine of Paris, etc., etc....359
The annual of scientific discovery; or year book of facts in science and arts. Edited by David Wells, of the Lawrence scientific school, Cambridge, and George Bliss....365
A universal formulary. By R. Eglesfeld Griffith, M. D....428
A practical treatise on inflammation of the uterus and its appendages, and on ulceration and induration of the neck of the uterus. By James Henry Bennet, M. D., M. R. C. P., &c....440
On the treatment of fractures, &c, by the use of the roller. By B. W. Dudley, M. D., professor of surgery in transylvania university....508
The diseases of females, including those of pregnancy and childbed. By Fleetwood Churchill, M. D., &c....524
Surgical anatomy. Part 2. By Joseph Maclise....525
A treatise on baths, &c. By John Bell, M. D., &c....527
Rupture of the ilium and omentum. By F. H. Gordon, M. D. ....................142
S
Salivation treated by nitrate of silver ....................266
Salt, experiments on the use of....532
Sanitary reform ....................367
Sciatic nerve, regeneration of....531
Scientific journals, postage on....183
Silliman's case of arsenical poisoning .................... 49
Silicic Acid....................196
Sneed on epidemic dysentery.... 1
St. Louis Medical Journal.......548
Summer instruction...............548
Surgery, Kentucky...............178
Surgery, American...............179
Swain's remarkable case of fracture of the skull................473
T
Tables for assisting beginners in the diagnostic application of morbid urine...............205, 208
Tetanus, traumatic case of.......317
The bandage in its application to fractures, &c.................. 92
The reviewer of Prof. Dudley's surgical essays................. 92
The reviewer of Prof. Dudley's essays ....................178
The diagnostic value of the urine. By E. B. Haskins, M. D.......185
The history, diagnosis, and treatment of edematous laringitis. By Elisha Bartlett, M D., professor of the theory and practice of medicine in the university of Louisville ....................209
The position of the medical profession in society ...............366
The Louisville medical school....269
The cause of cholera.............275
The history, diagnosis and treatment of fibrinous coagula of the heart. By D. W. Yandell, M. D..369
The bandage in the treatment of fractures, ulcers, gun-shot wounds, hemorrhage, &c., &c. By T. S. Bell, M. D............390
The causes of ulceration..........399
The acute ulcer..................403
The chronic ulcer................403
The irritable ulcer ...............404
The Buffalo Medical Journal.....459
The London Lancet, on fractures, hemorrhages, and gangrenous ulcers ....................477
The Ohio Medical and Surgical Journal....................544
Thersites, a professorial........453
Tilden & Co 's alcoholic extracts.275
Translation of the prog amme of the premium offered by the society of pharmacy, of Paris.....248
Traumatic tetanus, case of, by Dr. Emison ....................317
Treatment of diarrhea and dysentery of fever..................258
Treatment of fissures of the anus..264
Tumor malignant cured..........448
Two lectures on water. By F. H Gordon, M. D..............277
Typhoid fever in Alabama.........182
Typhus fever....................258
U
Ulcers, treatment of by the bandage ....................390
Ulcers, local applications..........390
Ulcers, the bandage commended for, by the great lights of the profession....................391
Ulcers, various methods of treating them................413, 414
Ulcers, old should they be healed?.422
Ulcers, gangrenous..............485
Urea, uric acid, &c..............194
Urinary sediments...............193
Urine diagnostic value of, by Dr. Haskins ....................185
Uterus, malformation of, morbid adhesion and partial osseous degeneration of the placenta.......300
W
Walker's fumigation for ulcers....415
Water, two lectures on, by F. H. Gordon, M. D.................277
Weissinger, G. W. obituary notice of....................276
Wells, Wm. Charles sketch of the life and character of, by Prof. E. Bartlett, M. D................ 22
Wendel, B. F. Dr. on purpura.... 13
Wound of the brain ..............245
Y
Yandell's D. W. cases at the Louisville marine hospital..........241
Yandell's, D. W. case of chronic pneumonia with anomalous physical signs..................312
Yandell on the diagnosis and treatment of fibrinous coagula of the heart....................369

### *Receipts of Medical Journal for May*, 1850.

| | |
|---|---|
| Dr. J. H. McKay, Big Spring, Ky., | $5 00 |
| H. M. Ward, Columbus, Ky., | 5 00 |
| E. B. Haskins, Clarksville, Tenn., | 10 00 |
| J. R. Duncan, Fountain Run, Ky., | 7 00 |
| A. J. Merrick, Freedom, Ind., | 5 00 |
| J. E. Shipp, Centreville, Tenn., | 5 00 |
| S. M. Hobbs, Mt. Washington, Ky. | 5 00 |
| J. Clayton, Christiansburg, Ky., | 5 00 |
| T. J. Crittenden, Ingram's Cross Roads, Ala., | 5 00 |
| C. J. Blackburn, Covington, Ky., | 5 00 |
| S. & B. Stubblefield, McMinnville, Tenn.. | 5 00 |
| W. J. Routh, Cassville, Mo., | 5 00 |
| H. J. Enders, Baton Rouge, La., | 5 00 |
| G. B. Miller, Fox, Iowa, | 10 00 |

---

### DELINQUENT SUBSCRIBERS.

This numerous class must see the necessity of making prompt remittances. We furnish a Journal which is not only a record of the progress of all the departments of Practical Medicine, but which contains the principles of practice adapted to the West and South. Its publication is attended with a heavy expenditure, and the subscribers should enable us to meet this expenditure without any trouble. We feel every assurance that we give them their money's worth, and we hope they will promptly respond to this call by remitting their dues.

PRENTICE & WEISSINGER, *Publishers.*

---

The Western Journal of Medicine and Surgery is published monthly by the undersigned, at the corner of Main and Fifth streets, Louisville, at $5 per annum, payable in advance. Each number contains 96 pages, making two volumes in the year of more than 550 pages each.

Contributions to its pages by the Physicians of the Valley of the Mississippi addressed tothe Editors, are respectfully solicited.

Letters on business, to be addressed, postage paid, to the publishers.

January, 1844. PRENTICE & WEISSINGER.

